# Bib Sign in Proximal Descending Thoracic Aorta Rupture on CT Angiography: Presentation of a Paradigmatic Case

**DOI:** 10.1155/2022/6947207

**Published:** 2022-12-05

**Authors:** Antonio Pierro, Alessandro Posa, Luca Iorio, Alessandro Tanzilli, Lucia Cucciolillo, Fabio Quinto, Mariacarmela Sciandra, Roberto Iezzi, Savino Cilla

**Affiliations:** ^1^Radiology Department, Cardarelli Regional Hospital, Viale Luigi Montalbò, 86100 Campobasso, Italy; ^2^Vascular Surgery and Endovascular Unit, Cardarelli Regional Hospital, Viale Luigi Montalbò, 86100 Campobasso, Italy; ^3^Department of Diagnostic Imaging, Radiation Oncology and Hematology, “A. Gemelli” University Hospital, L.go A. Gemelli 8, 00168 Rome, Italy; ^4^Ospedale L. Bonomo, Viale Istria, 76123 Andria, Italy; ^5^Medical Physics Unit, Gemelli Molise Hospital, L.go A. Gemelli, 1, 86100 Campobasso, Italy

## Abstract

Thoracic aortic rupture may present with subtle clinical and CT-angiography findings. Recognition of the imaging features of early rupture is key for timely diagnosis and treatment. This report presents a new sign of incipient proximal thoracic aortic rupture on CT-angiography.

## 1. Introduction

Aortic emergencies are a diagnostic and therapeutic challenge due to their life threatening potential if not promptly diagnosed and treated [1]. Acute aortic syndromes are the major nontraumatic component of aortic emergencies; they include penetrating atherosclerotic ulcer (PAU), intramural haematoma (IMH), and acute aortic dissection. All of those pathological entities share common clinical signs and symptoms, characterized by abrupt onset and severe back, chest, or abdominal pain [2, 3]. Diagnosis of thoracic acute aortic syndromes is mainly based on imaging; in particular, state of the art multidetector computed tomography angiography (CTA) is the gold standard for conclusive diagnosis and is fundamental for treatment planning [3, 4]. An optimized chest CTA scan protocol is mandatory to reduce pitfalls, to get to the right diagnosis, and effectively start the treatment of acute aortic disease [5]. ECG-gated CTA acquisition can improve image quality on the aortic root, where pulsation artifacts are very common and can mimic a dissection [6].

PAU represents 2-7% of acute aortic syndrome cases and is defined as an atherosclerotic lesion with an ulceration which penetrates the aortic innermost layer (tunica intima) and the internal elastic lamina; when the ulceration reaches the middle layer (tunica media) of the aortic wall, it determines blood accumulation and leads to IMH formation [7–10]. PAU increases the risk of aortic rupture up to 38% [11]. At CTA, PAU is characterized by an outpouching of contrast medium past the tunica intima of the aorta [12]. PAU is commonly located in descending aorta (60-70% of cases) and typically occurs in patients with atherosclerosis [13].

A common differential diagnosis for PAU is the ulcer-like projection (ULP), which represents a newly formed local disruption of the intima layer, with a wide connection to the aortic lumen, seen in IMH undergoing unstable evolution: the blood fills the IMH and enlarges it, increasing the risk of aortic dissection and rupture [3]; moreover, ULP can be also found in patients with no evidence of atherosclerosis [9]. At CTA, ULP pouch enhances to the same degree of the aortic lumen after contrast medium injection [14].

We present a case of acute aortic syndromes due to a PAU of the descending thoracic aorta, with associated acute IMH and superimposed ULP originating from the periphery of the PAU intimal entrance port.

## 2. Case Presentation

**Figure 1:**
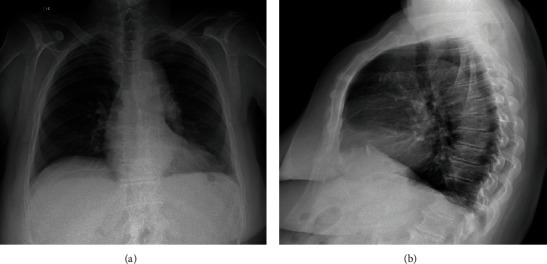
Chest radiograph in posteroanterior (a) and lateral (b) view: nothing abnormal detected.

A 77-year-old woman with a past medical history of uncontrolled hypertension, presented to the emergency room with 24 hours of unrelenting sudden-onset chest and back pain (Figure 1). Electrocardiography (ECG) showed normal recordings. The pretest clinical probability assessment according to aortic dissection detection risk score (ADD-RS) and D-dimer (DD) showed a value of ADD − RS > 1 (high risk) and DD value of >500 ng/mL [15]. In this scenario, the patient underwent urgent ECG-gated aortic CTA.

**Figure 2:**
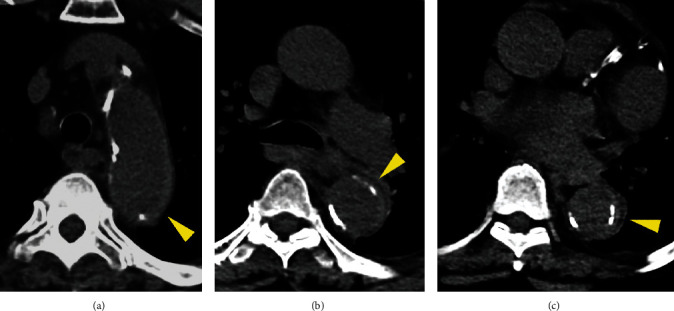
Unenhanced axial CT images of proximal thoracic aorta, showing a very thin aortic intramural hematoma below intimal calcifications (yellow arrowheads in (a–c)).

**Figure 3:**
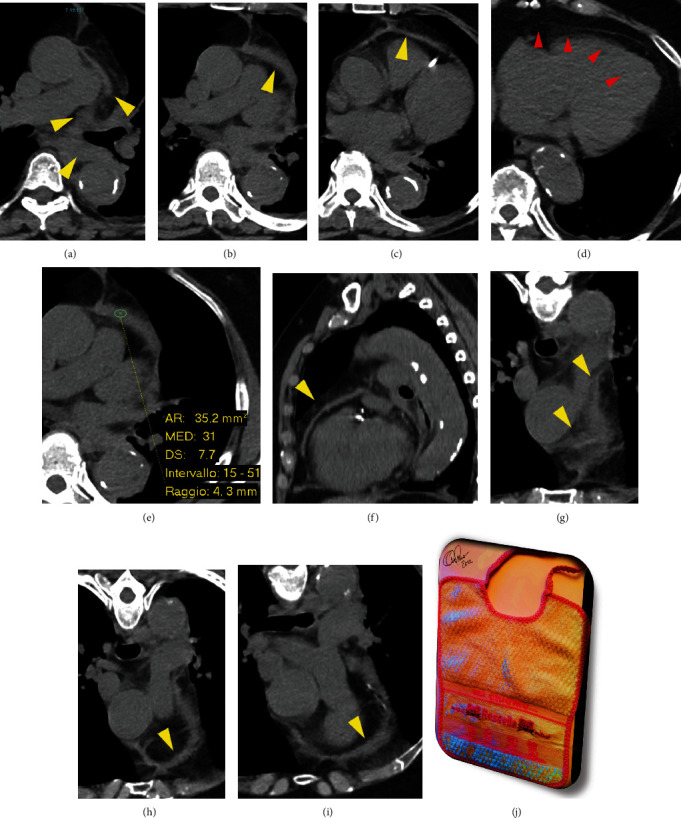
Unenhanced CT images showing the obliterated anterior descending aortic and paraesophageal fat due to hyperattenuating fluid collection (red arrowhead in (a)). The fluid flowed anteriorly through the extrapericardial inlet, under the left branch of the pulmonary artery, and above the left pericardial pulmonic recess of transverse sinus (green arrowhead in (a)). The fluid reached the anterior surface of the pericardial sac, between the prevascular and visceral mediastinal compartments (yellow arrowheads in (a–c) and (g–i)), and then flowed out caudally, covering the anterior wall of the right ventricle (yellow arrowheads in (f)), as a sort of “bib” on the heart (j). No associated pericardial effusion (red arrowheads in (d)). High-attenuation extrapericardial fluid (average 31 HU) consistent with hemomediastinum (e).

**Figure 4:**
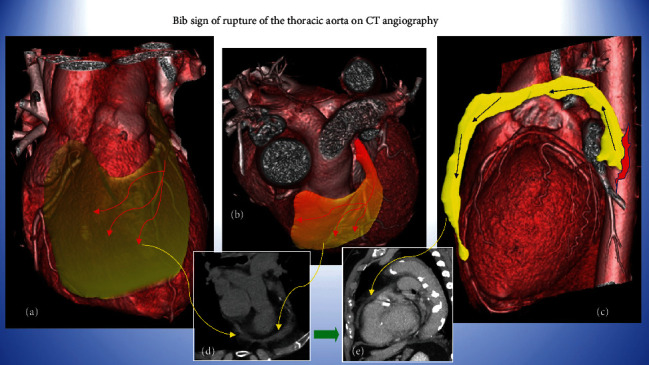
3D volume-rendering of the heart evaluated through various angles, demonstrating how the bib-like mediastinal hematoma is arranged and the path that it travels from the rupture site of the proximal descending thoracic aorta, up to the anterior surface of the heart (a–c). Corresponding CT images (d, e).

Unenhanced CT phase showed a thin, but extensive IMH propagating along the proximal thoracic aorta (Figure 2). A hyperattenuating fluid collection was also seen obliterating the periaortic fat tissue adjacent to anterior descending aorta and the paraesophageal fat tissue; the fluid collection flowed anteriorly, through the extrapericardial inlet, under the left branch of the pulmonary artery and above the left pericardial pulmonic recess of transverse sinus [16]. The fluid collection reached the anterior surface of the pericardial sac, between the prevascular and visceral mediastinal compartments, and then flowed out caudally, covering the anterior wall of the right ventricle as a sort of “bib” on the heart (Figure 3 and Figure 4) [17]. The hyperattenuating fluid flowing from the posterior visceral mediastinal compartment to the anterior prevascular mediastinal compartment was considered suspect for blood spread, with probable origin from the proximal descending thoracic aorta. There was no pericardial effusion, and the pericardium showed normal thickness.

**Figure 5:**
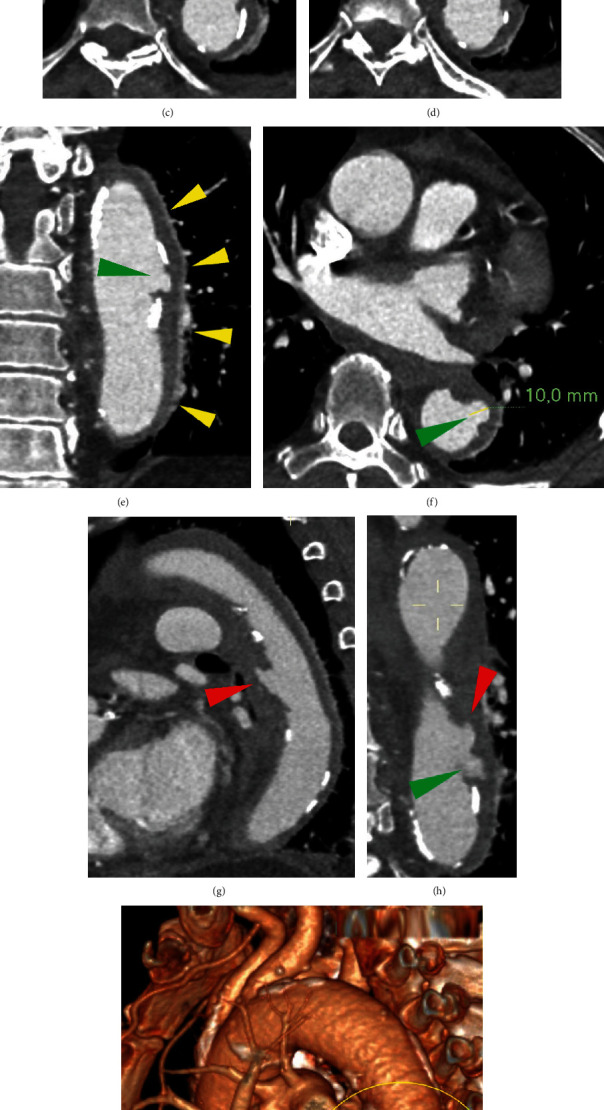
CTA showed a focal contrast-filled outpouching (PAU) of the proximal descending thoracic aorta (green arrowhead in (e, f, and h)), associated with a ULP originating from the entrance port of the PAU (yellow arrowheads in (b–d) and red arrowhead in g, h)). Obliterated paraesophageal fat (^∗^ in (a–d)) and aortic intramural hematoma are clearly visible (yellow arrowheads in (a)). Aortic 3D volume-rendering (i) and focal detail show a bifurcated aspect of PAU (green arrowhead) and ULP (red arrowhead) union.

CTA showed a focal contrast-filled outpouching of the proximal descending thoracic aorta, representing the PAU, associated with a ULP originating from the entrance port of the PAU, inside the IMH (Figure 5). On arterial and delayed CT phases, no macroscopic extravasation of luminal contrast media (arrow) was noted.

Symptomatic PAU and the associated ULP were treated with TEVAR, placing a RelayPro Thoracic Stent-Graft System (Bolton Medical) starting from under the aortic isthmus to the distal thoracic aorta. The descending aorta was not covered completely, to avoid the risk connected to Adamkiewicz artery occlusion.

**Figure 6:**
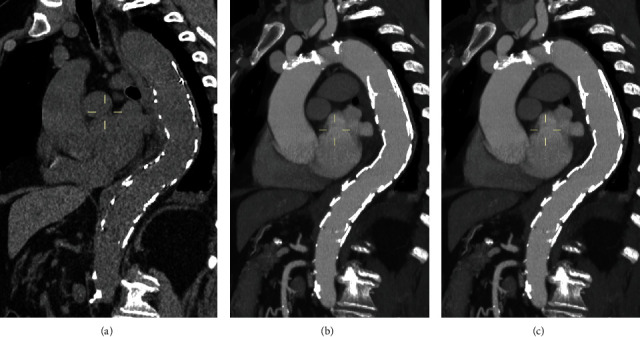
After 1 month, CTA reported a complete reabsorption of the IMH and a complete exclusion of the PAU and ULP (a–c).

The unrelenting chest and back pain vanished instantly after the TEVAR. After 1 month, CTA evaluation reported a complete reabsorption of the IMH and a complete exclusion of the PAU and ULP (Figure 6). Furthermore, the signs of mediastinal and paraesophageal hematoma were no longer detectable.

## 3. Discussion

Acute aortic syndromes are associated with high mortality and morbidity; it is mandatory to find and distinguish radiological features of acute aortic syndromes and rupture to guide the diagnosis and treatment. Imaging findings of thoracic aortic rupture seen at unenhanced CT include hemomediastinum (striped opacities or hyperdensity in the mediastinum), hemothorax, or hemopericardium; the hemorrhagic component measures more than 30 Hounsfield units (HU) in density and can be better detected at unenhanced CT scans. Contrast-enhanced CT scans help to identify the type of acute aortic syndrome and could show contrast medium extravasation [18].

In the reported case, the PAU is associated with a relatively extended IMH in a long segment of the descending aorta, and the IMH has been worsened by the subsequent formation of a ULP. All these pathological and symptomatic aortic entities were simultaneously present in our patient and required prompt treatment. To the best of our knowledge, ours is the first case of a symptomatic PAU-IMH associated with a ULP attached to the intimal opening of the PAU.

Furthermore, the presented case showed a mediastinal blood spread, exploiting the periesophageal and extrapericardial cardiac recesses (particularly the left pulmonic recess, under the left branch of the pulmonary artery), reaching the sloping portion of the interface between the visceral (posterior) mediastinum and the prevascular mediastinum (front). This CTA appearance, which we have called the “bib” sign, represents a sign that can be observed in the first moments of rupture (contained rupture) of the descending thoracic aorta. When this sign is observed at unenhanced CT examination, a possible cause of anterior wall rupture of the proximal descending thoracic aorta must be searched. In other words, the presence of the getaway offered by the extrapericardial recess below the left branch of the pulmonary artery allowed the initial blood spread not to form a periaortic hematoma in the virtual space anterior to the proximal descending thoracic aorta but to flow out anteriorly.

## Data Availability

Data sharing is not applicable to this article, as no new data were created or analyzed in this study.
